# Invasive Fungal Infections Associated with COVID-19

**DOI:** 10.3390/jof9060667

**Published:** 2023-06-14

**Authors:** Kyaw M. Hlaing, Lea M. Monday, Marcio Nucci, Simone A. Nouér, Sanjay G. Revankar

**Affiliations:** 1 Division of Infectious Diseases, Wayne State University School of Medicine, Detroit, MI 48201, USA; khlaing@med.wayne.edu (K.M.H.); lmonday@med.wayne.edu (L.M.M.); 2 University Hospital, Federal University of Rio de Janeiro, Rio de Janeiro 21941-853, Brazil; mnucci@hucff.ufrj.br (M.N.); snouer@hucff.ufrj.br (S.A.N.)

**Keywords:** aspergillosis, mucormycosis, candidiasis, COVID-19

## Abstract

The COVID-19 pandemic caused >6 million deaths worldwide, often from respiratory failure. Complications frequently occurred in hospitalized patients, particularly in the intensive care unit. Among these, fungal infections were a cause of high morbidity and mortality. Invasive aspergillosis, candidiasis and mucormycosis were the most serious of these infections. Risk factors included alterations in immune defense mechanisms by COVID-19 itself, as well as immunosuppression due to various therapies utilized in severely ill patients. Diagnosis was often challenging due to lack of sensitivity of current testing. Outcomes were generally poor, due to significant co-morbidities and delayed diagnosis, with mortality rates >50% in some studies. High index of clinical suspicion is needed to facilitate early diagnosis and initiation of appropriate antifungal therapy.

## 1. Introduction

Since the end of 2019, the novel severe acute respiratory syndrome coronavirus-2 (SARS-CoV-2) has caused a pandemic, with 573 million cumulative cases and reported cumulative deaths of 6 million until July 2022, according to the World Health Organization (WHO) [1]. The overall fatality rate was around 3.9% in 2020 (*n* = 680,894) [2]. While new therapies and vaccines are being developed, new variants are still forming and infecting people across the world daily.

According to a study by Rawson et al., the chance of bacterial and fungal coinfections is lower at the time of COVID-19 diagnosis but during hospitalization, the risk of acquiring co-infection increases, especially in critically ill patients requiring ICU admission and intubation [3]. In severe disease from COVID-19 and associated lung injury, the use of immunomodulators (baricitinib and tocilizumab) and immunosuppressants (dexamethasone) for cytokine storm syndrome predisposes patients to developing secondary coinfections including bacterial and opportunistic fungal lung infections (*Candida*, *Cryptococcus*, Mucormycosis, and *Aspergillus*) [4].

There are limited data and knowledge about these COVID-19-associated fungal infections, risk factors, immunogenicity, morbidity, mortality, and how to establish diagnosis and treatment [5]. Although COVID-19-associated fungal infection cases are uncommon, their morbidity and mortality are high, and with emerging antifungal-resistant fungi globally, management of these pathogens is complicated. We will review COVID-19-associated pulmonary aspergillosis (CAPA), invasive candidiasis, and pulmonary mucormycosis.

## 2. COVID-19 Associated Pulmonary Aspergillosis (CAPA)

Since the COVID-19 pandemic started, cases of COVID-19 associated pulmonary aspergillosis (CAPA) have risen. Pulmonary aspergillosis is caused by the fungus, *Aspergillus* and mainly by the species, *A. fumigatus*, and *A. flavus* [6]. Like influenza-associated pulmonary aspergillosis (IPAP), CAPA shows similar features that are associated with high mortality, especially in critically ill patients and immunocompromised hosts [7]. COVID-19 viral pneumonia causes lung epithelial damage and acute respiratory distress syndrome (ARDS) by dysregulation of the immunological response, and medications received during the cytokine storm syndrome all predispose patients to pulmonary aspergillosis [2,8]. Because of the limitations in data regarding epidemiology and diagnosis, many studies are still needed to improve our understanding of CAPA.

### 2.1. Epidemiology and Risk Factors of CAPA

Among hospitalized patients with COVID-19 infection, 14–38% of the patients developed severe respiratory distress and required intensive care unit (ICU) admission [9,10]. The incidence of CAPA is seen more in patients who are admitted to the ICU and subsequently require endotracheal intubation, with an incidence rate of about 20–35% [8,10]. According to Arastehfar et al., based on various European studies, the median days of onset for CAPA was 6 days after admission to the ICU, ranging from 3 to 28 days [11]. The major risk factors for CAPA are direct epithelial lung injury from COVID-19, dysregulation of the immune system and host factors which, in turn, damage the lung parenchyma, use of corticosteroids and immunomodulators therapies for cytokine storm syndrome; concurrent use of antimicrobial therapies for bacterial coinfection (azithromycin and beta lactam antibiotics) [11,12]; and comorbidities such as structural lung diseases, old age, morbid obesity, coronary artery disease, renal disease, and cancers.

Previously, invasive pulmonary aspergillosis (IPA) was commonly associated with hematologic diseases, malignancies, and conditions associated with severe immunosuppression such as acquired or congenital immunodeficiencies, chemotherapy and immunotherapy; hemopoietic stem-cell-transplantation; chimeric antigen receptor T-cell (CART) therapy; and long-term corticosteroid therapy. Other comorbidities which are associated with IPA are underlying chronic obstructive pulmonary disease, autoimmune diseases, liver cirrhosis, chronic kidney disease or renal replacement therapy, influenza infection, diabetes mellitus, and advanced solid organ cancers [13].

### 2.2. Immunopathogenesis of CAPA

Infection with SARS-CoV-2 can affect host immune defense mechanisms which protect against various pathogens including *Aspergillus*. Even without the traditional risk factors, patients with COVID-19 infection are still at a higher risk of developing CAPA, especially patients requiring intubation and ICU admission.

Upon entry of SARS-CoV-2 into the respiratory tract, it interacts with a specific receptor called angiotensin-converting enzyme 2 (ACE2) [14]. ACE2 is important for innate immunity and to balance the gut microflora. Despite the antiviral protection pathway to kill SARS-CoV-2, the virus still survives and triggers pro-inflammatory responses with subsequent cytokine storm syndrome.

Studies have demonstrated that COVID-19 infection significantly elevates interleukin-10 (IL-10) and interleukin-6 (IL-6) levels, which attracts other cytokines and chemokines such as TNF-α, interleukin-1β, and monocyte chemoattractant protein-1 [14,15]. IL-10 and IL-6 play key roles in the regulation of cellular immune responses and may induce cytokine release syndrome (CRS), respectively. An increased amount of IL-10 causes an influx of phagocytes into the tissue and can limit the fungal associated destruction of local tissue. An increase in Th-2 responses or decreased Th-1 responses causes the down-regulation of macrophages, and increases the host’s susceptibility to life-threatening invasive aspergillosis [14,15].

IL-6 is one of the main proinflammatory cytokines which has shown a protective effect against aspergillosis [15]. Although IL-6 has a protective effect, its excessive amounts during COVID-19 infection attract other cytokines and chemokines, leading to CRS which, in turn, causes increased vascular permeability, ARDS, and increased susceptibility to IPA [11]. During CRS, using a humanized monoclonal anti-IL-6 receptor antibody such as tocilizumab blunts the effect of IL-6 and improves oxygenation in CRS but may predispose to IPA. Therefore, anti-IL-6 can be used for COVID-19-related CRS but also increases the likelihood of acquiring IPA.

Interestingly, a commonly used antibiotic, azithromycin, influences neutrophil activation. Human trials have shown that azithromycin decreases serum IL-6, which induces delayed down-regulation of the neutrophil oxidative burst and increased neutrophil apoptosis. These factors are an important first-line defense against aspergillosis. This effect is seen when using 500 mg of azithromycin for more than 3 days and can last up to 28 days due to a long half-life [12]. Therefore, additional caution should be implemented while using azithromycin for secondary bacterial pneumonia, and steps should be taken to stop it as soon as possible when it is unnecessary.

### 2.3. Making Diagnosis of CAPA and Challenges

CAPA is defined as when IPA develops in the presence of positive COVID-19 RT-PCR within 2 weeks of hospital admission or within 72–96 h (about 4 days) from ICU admission. According to the 2020 European Confederation of Medical Mycology (ECMM)/International Society of Human and Animal Mycology (ISHAM) [16], the diagnosis of CAPA depends on host factors, and clinical/radiological and mycological evidence.

There are three subcategories in CAPA: proven, possible, and probable CAPA. Proven CAPA is diagnosed with histopathological or microscopical detection of *Aspergillus* invasively growing in lung tissues with resultant damage. Probable CAPA is defined as pulmonary infiltration, nodules, or cavitating infiltration documented by chest CT and mycological evidence obtained by bronchoalveolar lavage. Lastly, possible CAPA is defined as pulmonary infiltration, nodules, or cavitating infiltration documented by chest CT and mycological evidence obtained via methods other than bronchoalveolar lavage.

Host factors include previously discussed risk factors. The clinical features of CAPA are an intermittent fever of 3 days or more; a new fever after defervescence of longer than 48 h while on appropriate antibacterial drug therapy; deterioration in respiratory status; hemoptysis, pleuritic rub, or chest pain. Radiologic findings for IPA are mainly based on the CT scan and include a pulmonary infiltration with a halo sign, and nodular or cavitary lesions. CT scan findings should be accompanied by or supported with mycological evidence.

Making a definitive diagnosis of CAPA requires invasive bronchoscopy with bronchoalveolar lavage (BAL) specimens, but the procedure is associated with aerosol generation, exposing physicians and healthcare workers to the COVID-19 virus and may not be well-tolerated by patients in a critical condition [17,18]. Furthermore, access to bronchoscopies can be limited in low- to mid-level developing countries due to a lack of resources. Thus, serum tests and upper respiratory tract specimens such as endotracheal aspirates are more practical in diagnosing CAPA.

In non-invasive testing, galactomannan (GM) can be employed in both serum and BAL/non-BAL specimens. GM is measured using an optical density index (ODI) and it has a higher sensitivity of around 88–90% in BAL [6]. Previously for IPA, the cut-off level was >0.5 in BAL but recently with CAPA, an ODI level of >1.0 is preferred in some studies due to a higher specificity [6,11,19]. Although BAL GM is highly specific, serum GM has low sensitivity and when positive, may indicate angioinvasive aspergillosis [10,17,19]. Serum β-D-glucan can be used in the early stages of IPA and has been used as a means of screening. It has a higher sensitivity rate at 77% [6] and serial positivity increases the specificity and aids in supporting IPA diagnosis. There are still limited data using serum β-D-glucan in this setting. In European studies [6,10,11], an *Aspergillus*-specific lateral-flow device can be used in combination with serum β-D-glucan, and may increase the sensitivity from tracheal aspirate samples and result in high negative predictive value to rule out CAPA. Lastly, serum and/or BAL *Aspergillus* PCR may be useful in the diagnosis of IPA [7].

Even in the ICU setting, the diagnosis of IPA is still challenging and there is often a delay in diagnosis due to low sensitivity of non-culture-based tests. In addition, clinical and radiological features of IPA may be shared by other pathogens, including bacteria and non-*Aspergillus* fungi, or other conditions such as pulmonary edema and atelectasis, etc. [20]. The diagnosis may be established by postmortem biopsy though there is still risk in COVID patients of aerosol generation and exposure to health care personnel [21]. To mitigate this risk, in some centers, CT guided biopsies have been used as an alternative to autopsy to diagnose IPA. However, even this strategy may not be ideal. According to Flikweert et al., during the autopsies of six critically ill COVID-19 patients, there were no histopathological findings of CAP although there were high BAL GM test results pre-mortem [22]. In Rustsaert et al., there were also different results in GM based on the site of the samples such as serum vs. BAL. In this study, 20 critically ill COVID-19 patients were studied, and it was found that serum GM results were negative while BAL GM results were highly positive [23]. Thus, clinicians should use caution when interpreting GM results and need to correlate them with clinical and radiological features to more accurately diagnose CAPA.

### 2.4. Treatment and Preventive Measures

According to ECMM/ISHAM, voriconazole or isavuconazole for the treatment of possible, probable, or proven CAPA is the first-line therapy [11,16,18]. Voriconazole is a reliable drug for the treatment of IPA, but has some challenges in the treatment of CAPA in critically ill patients due to its narrow therapeutic level and multiple drug–drug interactions through CYP2C19, CYP2C9, and CYP3A4. It has also shown mild interaction with some COVID-19 treatments, such as remdesivir, which is also metabolized through CYP3A4 [11,16]. Compared to voriconazole, isavuconazole has fewer drug–drug interactions and has more favorable pharmacokinetics [11].

When triazoles (voriconazole or isavuconazole) cannot be used for the treatment of CAPA, liposomal amphotericin B is an alternative. The drawback of this medication is renal injury and in the setting of COVID-19 renal injury, it can worsen or cause acute kidney injury [11,16]. An alternative therapy for CAPA is posaconazole, which has multiple drug–drug interactions, as with other triazoles.

If azole-resistant *Aspergillus* is suspected, one can combine triazole therapy with echinocandins or use liposomal amphotericin B. Echinocandins are not considered a first-line therapy for IPA due to their limited antifungal activity against *Aspergillus* and fungistatic effect, but in combination with azoles, they have shown a synergistic effect and may overcome resistant strains [11]. Other newly developed antifungals might become future options in the treatment of CAPA but more data are needed to determine their effectiveness [11,16,18].

For the prevention of CAPA, Rutsaert et al. from the Netherlands have shown the benefits of prophylactic aerosolized liposomal amphotericin B in all COVID-19 patients on mechanical ventilators, and installing high-efficiency particulate air filters in the ICU [23]. There are ongoing trials for prophylactic posaconazole for severe influenza but data are still pending regarding its efficacy in CAPA prevention.

### 2.5. Mortality and Outcomes of CAPA

According to Mitaka et al. and Chong et al., the mortality of CAPA varies between 43–55%, even with appropriate antifungal therapy [8,19,21]. One study in Europe showed a 28-day mortality rate of 31% and a 90-day mortality of 37% in patients who were critically ill in the ICU with CAPA and requiring invasive mechanical ventilation [20]. Moreover, the time required to diagnose CAPA from COVID-19 symptom onset ranged from 8 to 16 days, compared to a CAPA diagnosis from ICU admission and after invasive mechanical ventilation ranging from 4 to 15 days and 3 to 8 days, respectively. The most common *Aspergillus* species identified was *Aspergillus fumigatus* [6,14,19]. According to Chong et al., the length of hospital stay was between 16 and 37.5 days, and the ICU stay ranged from 10.5 to 37 days. The duration of invasive mechanical ventilation with CAPA was 13 to 20 days [19]. Since CAPA cases are still rising across the globe, more studies and data are required to better understand these issues.

### 2.6. Invasive Candidiasis and COVID-19

The first reports of candidemia in patients with COVID-19 were published a few months after the beginning of the pandemic. In May 2020, three cases of candidemia were reported among 43 patients with COVID-19 who had been treated with tocilizumab [24]. Subsequently, a retrospective single-center study of bloodstream infections occurring in 78 critically ill patients with COVID-19 admitted to an ICU reported three cases of candidemia among 45 episodes of bloodstream infections [25]. In another publication, 15 cases of candidemia were diagnosed in 139 critically ill patients with COVID-19 (*Candida (C.) albicans* in nine, *C. parapsilosis* in four, and *C. glabrata* in two). All patients were under mechanical ventilation, vasopressor therapy, central venous catheters, parenteral nutrition, antibiotics, and corticosteroids [26]. A predominance of *C. auris* as the agent of candidemia in patients with COVID-19 was reported in a hospital in India: 15 cases of candidemia among 596 ICU patients (2.5%), 10 of which (67%) were caused by *C. auris*. All 10 patients had been in the ICU for a prolonged time (20–60 days) [27].

### 2.7. Risk Factors for Candidemia

As discussed in a review paper on COVID-associated candidiasis [28], the immunological alterations associated with COVID-19 (lymphopenia and activation of the innate immune system) are not associated with an increased risk of candidemia. On the other hand, critically ill patients with COVID-19 are exposed to various risk factors for candidemia, including the receipt of broad-spectrum antibiotics and corticosteroids, central venous catheters, dialysis, and parenteral nutrition [29,30]. These seem to be the main drivers of the occurrence of candidemia in critically ill COVID-19 patients.

An association between tocilizumab use and an increased risk of candidemia has been reported [24,31], but the topic is controversial. A study compared the characteristics of 80 patients with COVID-19 who were admitted to an ICU and developed candidemia with 160 controls (patients with COVID-19 in the ICU but without candidemia). Tocilizumab had been given to 41.3% of cases and 44.4% of controls (*p* = 0.65). By multivariate analysis, older age and a higher SOFA score were the only variables significantly associated with candidemia [32]. In contrast, in a study comparing 33 cases of COVID-19 and candidemia with 70 controls without candidemia, receipt of tocilizumab was associated with candidemia by multivariate analysis, with an odds ratio of 1.952. The other variables associated with candidemia in the multivariate analysis were prolonged hospitalization and high-serum D-dimer [33].

### 2.8. Increased Incidence of Candidemia

The first report of an increase in the incidence of candidemia in the COVID-19 pandemic was a single-center study conducted in Italy. The incidence of candidemia in patients with COVID-19 occurring between February and June 2020 was compared with the incidence in 2017 [34]. The incidence per 10,000 person-days of follow up was 10.97 in patients with COVID-19 compared with 1.48 overall, and 81.68 vs. 14.46 per 10,000 person-days of follow up in ICU patients.

Subsequently, we reported an increased incidence of candidemia with the COVID-19 pandemic in our center [35]. Historically, the incidence of candidemia in our center had been stable over a 21-year period, with an overall incidence of 1.3 episodes per 1000 admissions [36]. In the study, we compared the incidence of candidemia from January 2019 to February 2020 (pre-pandemic period) with the incidence from March to September 2020 (pandemic period). The overall incidence of candidemia in the two periods was 2.98 episodes per 1000 admissions, being 1.54 in the pre-pandemic period and 7.44 in the pandemic period (*p* < 0.001, risk ratio 4.83). The increased incidence was due to an increase in the absolute number of episodes of candidemia in the pandemic period but also due to a decrease in admissions in this period (723 monthly admissions in the pre-pandemic and 523 monthly admissions in the pandemic period). This reduction in admissions occurred because in preparation for the pandemic, the hospital administration implemented changes in the routine work, including cancellation of elective surgical and medical procedures, discharge of stable patients, deactivation of 50 regular beds, and the creation of two new areas for patients with COVID-19, including 25 beds for intensive care. We also observed that the higher incidence of candidemia in the pandemic period occurred not only in patients with COVID-19 but also in non-COVID patients, possibly reflecting the fact that during the pandemic period, only the most ill (and at higher risk for candidemia) patients remained in the hospital.

As previously mentioned, the study period of our paper was until September 2020 [34]. Following the waves of COVID-19 in Brazil, we continued to have a large number of cases of COVID-19 in our hospital until August 2021. After this period, the overall number of hospital admissions returned to values similar to pre-pandemic levels: 9060 in 2019, 6294 in 2020, 8690 in 2021, and 4191 in the first 6 months of 2022 (data not published). Likewise, with the dramatic reduction in hospitalizations for COVID-19, activity within the various specialties returned to a status similar to the pre-pandemic period. However, the incidence of candidemia (per 1000 admissions) continued to increase: 1.32 in 2019, 4.61 in 2020, 4.37 in 2021, and 5.01 in the first half of 2022. Species distribution also changed over the course of these 3.5 years. In 2020, *C. albicans* was the agent of 55% of episodes of candidemia, compared with 25% in 2019, 28% in 2021, and 22% in 2022. In contrast, *C. parapsilosis* was the agent of 48% of episodes of candidemia in 2022, compared with 25% in 2019, 10% in 2020, and 26% in 2021. Interestingly, an increase in the incidence of candidemia due to *C. parapsilosis* in the pandemic period has also been reported by other groups, including cases caused by fluconazole-resistant *C. parapsilosis* isolates [37,38,39].

### 2.9. Clinical Characteristics

We compared the characteristics of 9 episodes of candidemia in patients with COVID-19 with 16 occurring in patients without COVID-19 diagnosed in the same period [35]. All patients with COVID-19-associated candidemia were under mechanical ventilation, compared with 34.4% of non-COVID-19 patients (*p* < 0.001). Likewise, COVID-19 patients were more likely to be in an ICU (77.8% vs. 40.6%, *p* = 0.07) and to be on vasoactive drugs at diagnosis of candidemia (88.9% vs. 50.0%, *p* = 0.06), and less likely to have surgery within 30 days before candidemia (0 vs. 31.2%, *p* = 0.08).

A case-level analysis of candidemia using population-based surveillance data conducted in the USA compared the characteristics of candidemia in patients with or without COVID-19 [29]. Among 251 cases of candidemia, 64 cases occurred in patients with COVID-19 and 187 cases occurred in patients without COVID-19. Candidemic patients with COVID-19 were older, more likely to have diabetes, obesity, to be in ICU in the previous 2 weeks, to be under renal replacement, mechanical ventilation, and to have received corticosteroids or tocilizumab. In contrast, candidemic patients without COVID-19 were more likely to have gastrointestinal disease, liver disease, solid tumors, to have undergone surgery in the previous 3 months, and to be under total parenteral nutrition. Another important difference between cases of candidemia with or without COVID-19 was the time of occurrence of candidemia since hospital admission: a median of 4 days in cases without COVID-19 and 14 days in cases with COVID-19. On the other hand, no differences in *Candida* species distribution were observed. The mortality rate was significantly higher in cases of candidemia associated with COVID-19 (62.5% vs. 32.1%, *p* < 0.001), though management is similar to cases without COVID-19.

## 3. COVID-19-Associated Mucormycosis (CAM)

Mucormycosis is an opportunistic, angio-invasive, life-threatening infection caused by fungi of the class, Zygomycetes. The majority of human disease due to Zygomycetes is caused by the Mucorales order. Mucorales include a variety of thermotolerant fungi found ubiquitously throughout the world in decaying matter and soil, and varied in distribution by geographic location [40]. Common genera include *Rhizopus*, *Mucor*, *Rhizomucor*, *Lichtheimia* (formerly *Absidia)*, *Apophysomyces*, and *Cunninghamella*, with the most common causative species globally being *Rhizopus arrhizus* [40,41]. As a human infection, mucormycosis is divided clinically based on the anatomical site of infection. The most common form is rhino-orbito-cerebral mucormycosis (ROCM) which encompasses a spectrum ranging from limited sino-nasal disease to that progressing into the orbits and brain. Mucormycosis can also present as pulmonary, cutaneous, gastrointestinal, renal, and disseminated forms [42]. In late 2020, the U.S. Center for Disease Control and Prevention released its first report on COVID-19-associated fungal infections, including aspergillosis and candidiasis [43]. Reports of COVID-19-associated mucormycosis (CAM) began to increase in mid-2021, when the Delta variant triggered a second COVID-19 wave in India [44,45,46,47]. CAM is defined as a microbiologically or histologically confirmed case of mucormycosis, diagnosed during or after confirmation of SARS-CoV-2 infection [45,46,47,48]. The timeline for how long after a COVID-19 diagnosis one would define the mucormycosis as CAM varies, with some sources citing up to 60 days after, and others up to 90 days [46,47,48].

### 3.1. Epidemiology and Risk Factors

Prior to COVID-19, the prevalence of mucormycosis varied globally from 0.005 to 1.7 per million population, but was nearly 80 times higher in India compared to developed countries [41,48]. The SARS-CoV-2 Delta variant triggered a second wave of COVID-19 on the Indian subcontinent in mid-2021. Subsequently, the prevalence of mucormycosis in Indian centers doubled, leading to an acute shortage of amphotericin B and the Indian government designating mucormycosis as a notifiable disease [49]. Additional cases of CAM have since been reported across the world, including centers in the United States, Mexico, and Europe [50,51].

Mucormycosis is known to occur in hosts with uncontrolled diabetes or other immunosuppressive risk factors such as high-dose corticosteroids, chemotherapy, immunotherapy, prolonged neutropenia, or those with stem cell and solid organ transplants [52]. The ROCM form is frequently observed in association with uncontrolled diabetes and DKA, whereas pulmonary involvement is more often observed in patients with neutropenia, bone marrow and organ transplants, and hematological malignancies [48,52]. However, in the early cohorts of CAM emerging from India, few patients were noted to have the traditional risk factors for invasive mold infections seen in the developed world such as hematologic malignancy and solid-organ transplant [44,45,46]. The higher overall prevalence of CAM in low- and middle-income countries including India is thought to be driven by a high prevalence of undiagnosed diabetes mellitus, combined with excessive corticosteroid use and environmental conditions which favor a high baseline prevalence of mucormycosis [44,45,46,47,48]. Crowding, multigenerational housing, and shortages of resources contributed to the rapid uncontrolled spread of COVID-19 in such countries, though mucormycosis itself is not spread via human-to-human transmission.

### 3.2. Immunopathogenesis

Hyperglycemia is the most common risk factor for mucormycosis, both CAM and non-COVID-19-related forms [53,54]. Stress-induced hyperglycemia attributable to cortisol and pro-inflammatory cytokines is noted in patients with acute illness, including up to 50% of patients hospitalized with severe or critical COVID-19 [55]. Diabetes and hyperglycemia impair innate immunity by decreasing phagocytic function and are known risk factors for increased disease severity in COVID-19 [53,54]. Additionally, hyperglycemia causes glycosylation of transferrin and ferritin, leading to increased free iron as a resource for uptake by mucormycosis [56]. SARS-CoV-2 itself can also damage pancreatic islet cells, where it gains entry via the ACE2 protein receptor [53,54]. Thus, COVID-19 worsens glycemic control, and patients with poor glycemic control at baseline are more prone to severe COVID-19.

The role of corticosteroids in CAM has also been studied. In one early multicenter study, corticosteroids were used inappropriately in 63% of patients with CAM [44]. This was compounded by their over-the-counter availability and a national shortage of oxygen in India resulting in indiscriminate use including in patients unaware they had diabetes. Corticosteroids increase blood glucose levels and insulin resistance, directly increasing the major risk factors for CAM discussed above. Although they are beneficial for the immune dysregulation and inflammation that occurs in COVID-19, corticosteroids have broad and indiscriminate immunosuppressive effects. Even short courses of corticosteroids have been reported to be associated with mucormycosis, especially in subjects with diabetes [57,58].

Other factors related to the immunopathogenesis of CAM include endothelial damage and overexpression of the glucose-regulated protein called GRP78. GRP78 is expressed on the surface of mammalian endothelial cells and serves as the attachment for fungal ligand spore coating homolog (CotH) proteins which enables endocytosis and angio-invasion [48,58]. The exact pathogenesis behind CAM remains unknown as the effects of SARS-CoV-2 on innate immunity continue to be explored.

### 3.3. Diagnosis and Treatment

In the present CAM epidemic, most cases (90%) have presented as ROCM and the rest mostly as pulmonary or disseminated disease [44,45,46,47,48]. Signs and symptoms of ROCM include facial pain, nasal congestion, bloody or brown nasal discharge, sinus tenderness, fever, nausea, and headache [59]. Symptoms may overlap with those of COVID-19 and thus, careful physical examination including the palate, cranial nerves, eyes, and sinuses as well as a high index of suspicion is critical [51,52]. Complete details on the diagnosis of mucormycosis have been given by Cornely et al. in the ECMM/MSG worldwide guidance [60]. For diagnosing CAM, SARS-CoV-2 infection should be confirmed by a positive PCR or antigen test along with the clinical, radiographic, histopathologic, or mycologic evidence of mucormycosis [52]. If ROCM is suspected, patients should undergo imaging of the brain and sinuses, including computed tomography (CT) or magnetic resonance imaging (MRI). Further pulmonary imaging should be obtained if there is suspicion of pulmonary CAM, though features such as the reverse halo sign, nodularity, or cavitation are non-specific and overlap with other fungal co-infections including aspergillosis/CAPA [52]. A mycologic diagnosis can be made by examination of tissues taken via sampling of the suspected anatomic site. Tissue or biopsy samples should be first examined with KOH or calcofluor-white to identify characteristic aseptate ribbon-like hyphae with right-angle branching. Samples should not be crushed or ground as this can damage the fragile cell wall of *Mucorales* and decrease culture sensitivity and histopathologic accuracy [52,60]. There are no biomarkers specific to mucormycosis; however, negative aspergillus galactomannan antigen, 1,3-β-D-glucan, and *Aspergillus* PCR results in a patient with suspected invasive fungal infection of the lungs may suggest pulmonary mucormycosis [60].

The clinical management of CAM is similar to that of mucormycosis in non-COVID-19 patients. The full management of mucormycosis is further outlined in the ECMM-MSG global guidelines [60]. Three principles are key to management, which include controlling the underlying risk factor, early and aggressive surgical debridement, and initiation of antifungals. Comorbid conditions and controllable risk factors such as hyperglycemia and ketoacidosis should be controlled aggressively and immunosuppressant medications should be reduced or eliminated if feasible. Primary antifungal prophylaxis is not recommended [52]. Antifungal therapy with amphotericin B (ideally liposomal amphotericin B at a dose of 5 mg/kg per day in 200 mL of 5% dextrose) should be started as soon as possible [52,60]. Delay in the initiation of amphotericin of ≥6 days from presentation has been shown to double mortality in patients with hematologic malignancy and mucormycosis [61]. How a delay in amphotericin treatment may impact CAM patients remains unknown. Extended spectrum azoles (isavuconazole and posaconazole) are newer alternatives to amphotericin B; however, some *Mucorales* respond poorly to azoles in vitro and amphotericin B remains the optimal empiric treatment. The *Mucorales* are intrinsically resistant to voriconazole, fluconazole, caspofungin, anidulafungin, micafungin, and 5-fluorocytosine; these drugs should not be used for empiric treatment. Treatment strategies for when amphotericin formulations are not available due to shortage are discussed elsewhere [52]. Combination antifungals (amphotericin B with an extended spectrum azole such as isavuconazole or posaconazole) have been associated with better survival in some early case series [44]; however, combination therapy is not currently recommended [52]. Early and aggressive surgical debridement is equally crucial [60]. In a recent review of 80 CAM cases across 18 countries, 45 (58%) patients underwent surgical resection [51]. The ROCM patients without progression to CNS involvement who underwent surgery had improved outcomes compared to those who were treated with antifungals alone (mortality of 14% versus 63%, *p* = 0.012).

### 3.4. Mortality and Outcome

All-cause mortality in patients with CAM cases series has mostly been reported in the range of 25–49% [44,46,47,48,51]. Outcomes have varied depending on the form of mucormycosis and the country of the study. CAM due to pulmonary or disseminated disease has been identified as a risk factor for increased mortality compared to ROCM in at least two studies including mostly patients from India [46,51]. In a multinational review including 80 CAM patients, all-cause mortality was lower in patients with ROCM compared to pulmonary, gastrointestinal, or disseminated mucormycosis (37% versus 81%, respectively) [51]. Of the 59 patients included with ROCM, mortality was higher once disease progressed to confirmed CNS involvement (59% versus 24%, respectively). Life-altering morbidities were seen in those who survived, including loss of vision in 46% [51]. In another small series of 10 CAM patients in the United States (notably only four of whom had ROCM and the rest had either pulmonary, disseminated, or gastrointestinal disease), mortality was 60% [50]. These differences in mortality are consistent with the heterogeneity in patients, and clinical forms of illness being studied. Overall, studies in Indian cohorts, which consist mostly of patients with ROCM who tend to be younger with undiagnosed diabetes, differ significantly from a US or European cohort which tend to include more elderly patients with organ transplant or hematological malignancies and include a higher proportion of pulmonary, disseminated, and gastrointestinal disease.

COVID-19-associated mucormycosis is a serious complication of severe COVID-19, particularly in patients with uncontrolled diabetes and hyperglycemia due to corticosteroids and severe illness. Diagnosis is challenging and requires a high degree of suspicion, particularly for pulmonary forms where imaging may overlap with COVID-19 or other fungal co-infections such as aspergillosis. Treatment should focus on control of the underlying risk factors, prompt surgical debridement, and amphotericin B therapy. Mortality rates vary depending on the anatomic form of the disease and on patient population, but are generally highest in patients with pulmonary or disseminated disease, or for those with ROCM which has progressed to cerebral involvement.

## 4. Summary

Invasive fungal infections associated with COVID-19 are an increasing problem in hospitalized patients. Risk factors are often related to typical nosocomial interventions such as intravenous catheters and broad-spectrum antibiotics in the case of candidemia in ICU patients, and significant immunosuppression associated with use of steroids in hospitalized patients with COVID-19 in the case of invasive mold infections, including aspergillosis and mucormycosis. In addition, immune dysfunction related to COVID-19 may also play a role, particularly in severe cases. Diagnosis is a challenge, requiring a high degree of suspicion in at-risk patients. Therapy generally follows standard recommendations for these infections, though mortality may be higher, especially in critically ill patients. Additional work is needed to elucidate strategies to improve early diagnosis and institute appropriate therapy.

## Data Availability

No new data were created or analyzed in this study. Data sharing is not applicable to this article.
